# Density-mediated foraging behavioral responses of *Rhyzopertha dominica* (Coleoptera: Bostrichidae) and *Tribolium castaneum* (Coleoptera: Tenebrionidae)

**DOI:** 10.1038/s41598-024-62277-8

**Published:** 2024-05-28

**Authors:** Marco A. Ponce, Sabita Ranabhat, Alexander Bruce, Taylor Van Winkle, James F. Campbell, William R. Morrison III

**Affiliations:** 1 Department of Entomology, Kansas State University, 123 Waters Hall, 1603 Old Claflin Place, Manhattan, KS 66506 USA; 2 Department of Entomology and Plant Pathology, University of Tennessee, 2505 E. J. Chapman Dr., 370 Plant Biotechnology, Knoxville, TN 37996 USA; 3 School of Planning, Design, and Construction, Michigan State University, East Lansing, MI 48824 USA; 4 Agricultural Research Service, Center for Grain and Animal Health Research, USDA, 1515 College Ave., Manhattan, KS 66502 USA

**Keywords:** Lesser grain borer, Red flour beetle, Stored products, Semiochemicals, Taxis, Integrated pest management, Ecology, Behavioural ecology

## Abstract

*Tribolium castaneum* and *Rhyzopertha dominica* are cosmopolitan, destructive postharvest pests. Although research has investigated how high densities of *T. castaneum* affect attraction to the aggregation pheromone by conspecifics, research into the behavioral response of both species to food cues after high density exposure has been lacking despite its importance to foraging ecology. Our goal was to manipulate and observe the effects of crowding on the behavioral response of both species to common food and pheromonal stimuli and to determine how the headspace emission patterns from grain differed under increasing densities. Densities of colonies for both species was altered (10–500 adults) on a fixed quantity of food (10 g of flour or whole wheat), then the behavioral response to common food and pheromonal cues was evaluated in a wind tunnel and release-recapture experiment, while volatiles were examined through gas chromatography coupled with mass spectrometry. Importantly, at least for *T. castaneum*, crowded conditions attenuate attraction to food-based stimuli, but not pheromonal stimuli. Crowding seemed to have no effect on *R. dominica* attraction to food and pheromonal stimuli in the wind tunnel, but exposure to high density cues did elicit 2.1–3.8-fold higher captures in traps. The relative composition and abundance of headspace volatiles emitted varied significantly with different densities of beetles and was also species-specific. Overall, our results have implications for expanding our understanding of the foraging ecology of two economically important pests.

## Introduction

Much effort over the last century in ecology has been devoted to studying the effect of population density on populations. Density often negatively affects the growth rate of populations^1^, while it may modulate other key processes such as, behavior^2^, optimal foraging^3^, host-parasitoid interactions^4^, and insect-mold interactions^5^. Moreover, density along with trait-mediated indirect interactions, have both been found to contribute to structuring communities, leading to the coexistence of insect species^6^. As a species approaches its carrying capacity in an ecosystem, there may be several patterns that emerge. This may include an increase in avoidance behavior and decrease in joining behavior by individuals to a group^7^, differences in optimal foraging behavior^8^, changes in predation^9^, modulation of chemosensation^10^, as well as increasing intra- and interspecific competition^11^.

Prior to reaching high density situations (or after high density situations), a key challenge faced by individual insects is locating food patches at a distance typically through volatile organic compounds (VOCs). During this process, individuals must assess the quality of a patch, and determine when to leave or how long to stay. In some cases, early arrivals may recruit conspecifics that may arrive *en masse*, feed, and sometimes mate, as in the case of bark beetles^12^ and some stink bugs^13^. The density of conspecifics in the environment may affect volatile emissions by conspecifics^14^. At high densities, some species may emit anti-aggregation pheromones^14^ or alarm pheromones^15^ to disperse individuals. Some male broad-horned flour beetle, *Gnatocerus cornutus* (F.) (Coleoptera: Tenebrionidae) respond to male-perfumed females or increased presence of rival males by allocating more effort to sperm competition^16^. In another case, researchers found a density–dependent polyphenism in pathogen resistance to entomopathogenic fungus and immune function in the mealworm, *Tenebrio molitor* L. (Coleoptera: Tenebrionidae)^17^. However, there have been few studies evaluating how an experience in a highly dense environment may then affect subsequent foraging decisions in an environment that is no longer densely populated.

Much work has been done on the foundational movement and dispersal ecology of red flour beetle, *Tribolium castaneum* (Herbst) (Coleoptera: Tenebrionidae) and lesser grain borer, *Rhyzopertha dominica* (F.) (Coleoptera: Bostrichidae). This makes them particularly good models to evaluate density-mediated effects under controlled conditions that nonetheless reflect the real-world conditions they may encounter. In addition, *T. castaneum* has been used as a model species for at least the past five decades^18,19^ was the first beetle pest to have its genome sequenced^20^, and is easy to handle and manipulate for experiments^21^. Each species has significantly different life histories, with *T. castaneum* playing a role as a secondary pest feeding and reproducing on damaged or processed products^22^, and *R. dominica* attacking and ovipositing in intact whole kernels^23^. The male-produced aggregation pheromone for *T. castaneum* consists of four stereoisomers of (4*S*/*R*, 8*S*/*R*)-4,8-dimethyldecanal^24^ and is attractive to both sexes, while for *R. dominica* it is comprised of the male-produced stereoisomers, (*S*)-( +)-1-methylbutyl (*E*)-2-methyl-2-pentenoate and (*S*)-( +)-1-methylbutyl (*E*)-2,4-dimethyl-2-pentenoate, and is also attractive to both sexes^25,26^. Each species has different movement and dispersal patterns. For example, *R. dominica* are considered weak walkers but once they reach the adult stage, they are strong dispersers via flight, capable of immigrating between agricultural and non-agricultural landscapes^27–30^. By contrast, *Tribolium castaneum* adults are recognized to be strong walkers^31^, but have weaker dispersal by flight; typically, conspecifics are primarily found in food facilities and adjacent areas^32–34^. As a result, each species may respond differently to density-mediated pressures.

Prior work has already evaluated some density-mediated effects on the response and life history of *T. castaneum*. For example, it is known that increased density slowed down the development rate and decreased body weight of *T. castaneum*^35^, but did not appear to significantly increase flight initiation or change time to initial flight by conspecifics^36^. Generally, once a stored product insect finds a new suitable food patch, an aggregation pheromone will be released to attract conspecifics or a sex pheromone will be released by one sex to attract the opposite sex^37^. However, under high density situations some stored product insects will employ density-mediated stress cues that halt the release of aggregation pheromone and induce conspecifics nearby to disperse^38^. This may function to avoid competition and increase the survival rate. Density-mediated cues produced by *T. castaneum* include a variety of methyl- and benzoquinones, which function as anti-aggregation pheromones, causing beetles to rapidly disperse^14,39^. Prior work has found that density affects the release of these volatiles by *T. castaneum*, as well as the behavioral response of conspecifics to the presence of the aggregation pheromone^14^. Natal habitat experience was found to modulate density-dependent dispersal by *T. castaneum*^40^, suggesting an interaction between habitat cue perception and density.

There has also been prior work evaluating some density-mediated effects on the response and life history of *R. dominica*. For example, *R. dominica* of multiple strains (field and laboratory) reared under crowded conditions had significantly higher flight initiation than conspecifics reared in uncrowded conditions^41^. Indeed, while *R. dominica* reared in uncrowded cultures had a low flight response, the number of beetles initiating flight increased with exposure to increased concentrations of frass or uric acid from conspecifics^42^. In addition, with increasing density, aggregation behavior by *R. dominica* decreased in a grain bulk containing wheat or maize^43^. Prior work has shown that reducing the density of males per quantity of food, individual males release more pheromone than controls and are a stronger signal for responders^44^. Additionally, that same study found the quantity of pheromone emitted per male declined with increasing beetle density and that this effect is stronger in the presence of other males than of females. Thus, while quite a bit is known about how density affects the life history of both *T. castaneum* and *R. dominica*, it is unknown how extended experience in a high density environment affects later semiochemical-mediated foraging choices.

One of the key cues used by stored product insects in foraging are food cues from post-harvest commodities (reviewed in 45). For example, prior work has found that host cues from grain are broadly attractive to a range of stored product insects, including *T. castaneum* and *R. dominica*^45–50^. Out of seven plant species, *R. dominica* was most attracted to wheat, which was found to be most suitable for its development^51^. However, without presence of pheromone^52^, response to food cues may sometimes be limited by both *T. castaneum* and *R. dominica*. However, how prior experience with high density conditions affects the response of stored product insects to these and other food cues, and thus host-finding, is not well understood but can have significant impacts on populations and pest monitoring programs. Thus, the aim of this study was to elucidate the effects of density (e.g., crowding) on the behavioral response of *R. dominica* and *T. castaneum* to common food-based attractants and pheromonal stimuli. In order to accomplish this, the density of individuals for a species was varied between 10 and 500 adults for four weeks, and then their behavioral responses to a variety of food cues were observed. We assessed the behavioral responses of insects using wind tunnel and release-recapture assays. In addition, we also characterized how the volatile emissions patterns from grain with beetles of each species at various densities differed in order to identify potential cues that are used by these species to modulate dispersal and volatile response behaviors. The volatiles from beetles at different densities were characterized using a headspace collection system, and then examined through gas chromatography coupled with mass spectrometry (GC–MS).

## Materials and methods

### Colony maintenance and experimental insects

Beetles used in this study were obtained from stock colonies kept in the laboratory of the USDA situated in Manhattan, KS. Colonies of *T. castaneum* (strain from eastern KS, collected in 2012) were reared on 95% organic unbleached flour and 5% brewer’s yeast, while *R. dominica* (strain from outside a mill in central KS, collected in 2012) colonies were cultivated on organic whole kernel wheat. For both species, colonies were initially subcultured with 2- to 4- wk-old adults to create density treatments below, which were kept in 950-mL mason jars (8.5 D × 17 cm H) and stored in an environmental chamber (136VL, Percival Instruments, Perry, IA, USA) set at constant conditions (27.5 °C, 60% RH, and 14:10 L:D).

### Density treatments

To examine the effects of density on the behavioral response of *T. castaneum* and *R. dominica* to common food and pheromonal semiochemicals, adults 4- to 8-wk post-emergence of both species were removed from stock colonies described above and reared in subcolonies at different densities. Beetles of 1:1 sex ratio (*T. castaneum*) or mixed sex (*R. dominica*) were placed in glass containers (5 cm D × 6.6 cm H) with 20 g of 95% organic flour + 5% brewer’s yeast (*T. castaneum*) or organic whole kernel wheat (*R. dominica*). A total of 10, 50, 100, or 500 individuals per container were used for *T. castaneum*, while 10, 50 or 100 individuals per container were used for *R. dominica*. Because of limitations with colony populations, 500 individuals per container were not used for *R. dominica* in the experiments below. The control consisted of food only without beetles, and was the source of uncontaminated grain or flour described below. The beetles of both species were held under each density for 3–4 weeks before use in experiments described below.

### Food & pheromonal semiochemicals

In the assays described below, we assessed the response of beetles to six semiochemical treatments that contained food stimuli alone or a combination of pheromonal and food stimuli (Table 1). Stored product beetle tab lures (SPB lures), wheat germ oil (WG), and Trécé Storgard Oil (TSO) was purchased in May 2018. Fresh samples of semiochemicals were used for each day of testing.

**Table 1:** List of food and pheromone semiochemical treatments used.

Abbreviations	Semiochemical treatment	Amount in wind tunnel	Amount in release-recapture	Function	Source	
Ctrl	Control	Empty	Empty	Unbaited control		
WG	Wheat germ oil		950 μl	950 μl	Food stimuli	
CF	Contaminated food	20 g	6 g	Food stimuli + potential repellents + attractants	Wheat flour or wheat kernels previously exposed to beetles: *T. castaneum* 500-beetle (flour + yeast) or *R. dominica* 100-beetle (whole wheat) densities for 3–4 weeks with beetles removed	
UCF	Uncontaminated food	20 g	6 g	Food stimuli	Analogous commodities to CF without exposure to beetles	
TSO	Trécé Storgard oil	950 μl	950 μl	Food stimuli	Trece, Inc., Adair, OK, USA	
SPB	Stored product beetle tab lure	1 lure	1 lure	Food stimuli + pheromone	Insect Limited,Westfield, IN, USA	

### Wind tunnel assay

**Figure 1:**
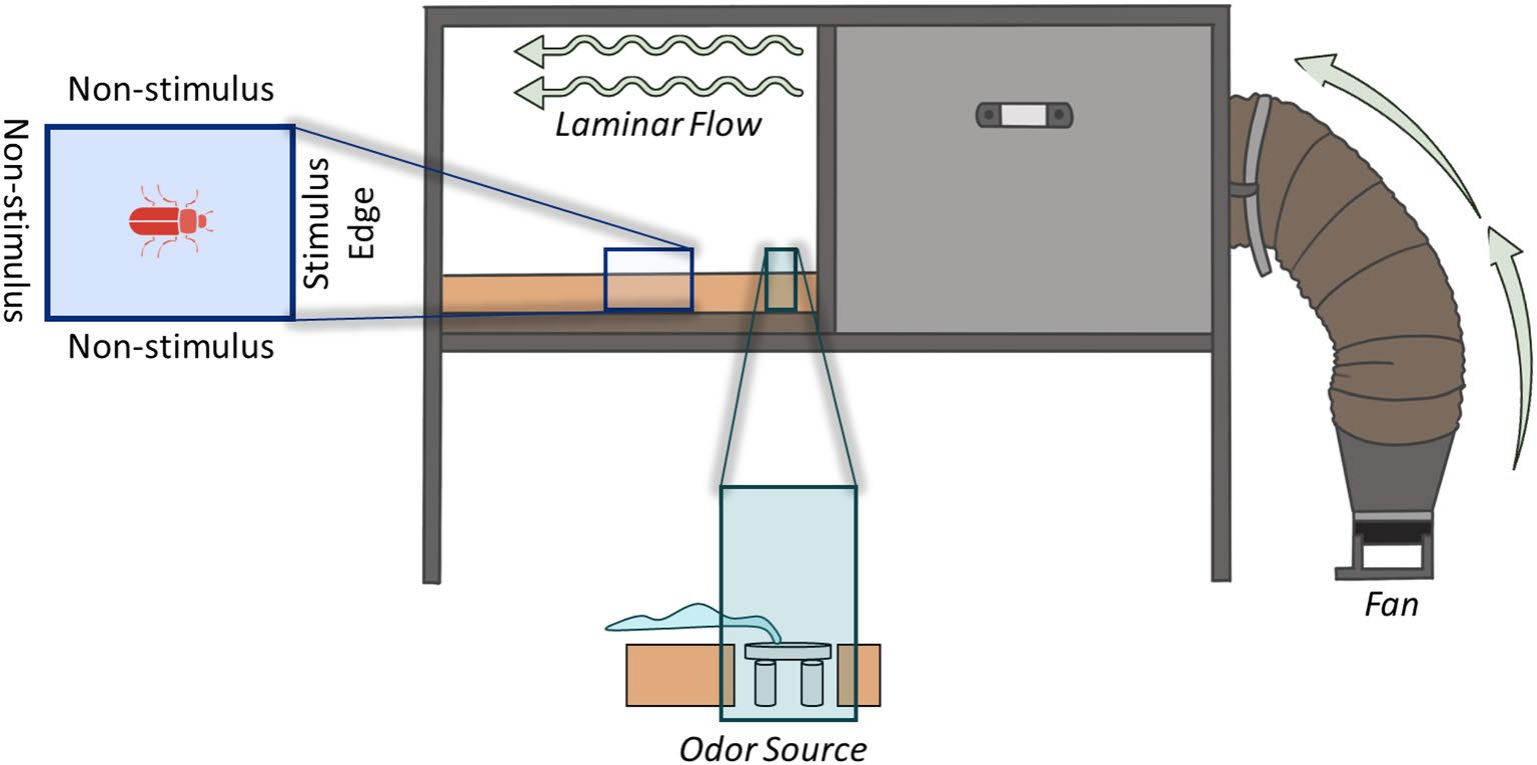
Schematic of wind tunnel experimental setup in the laboratory under controlled conditions in a walk-in growth chamber.

A laminar flow wind tunnel (94.5 × 73 × 80.5 cm L:H:W) was used to assess walking locomotory upwind orientation of *T. castaneum* and *R. dominica* that had been held at different densities, to food and pheromonal semiochemicals (Table 1)*.* This is a common quick assay to assess horizontal locomotory response of stored product insects to stimuli^53,54^. The wind tunnel consisted of an electric fan, which pushed ambient air at 0.39 m/s, first through two metal sieves to straighten airflow, and then an activated carbon filter to purify the air (Fig. 1). The semiochemicals (as described above) were placed 5 cm downwind of the last sieve in a 100 × 15 mm plastic petri dish flush with the surface of the orienting beetle, and 13.5 cm upwind of the release arena. Each release arena consisted of a 21.6 × 27.9 cm piece of paper and was changed between trials to prevent cross-contamination by trace chemical stimuli deposited by beetles or the various treatments. A smoke test confirmed that the air flowing over the Petri dish reached the release arena. In each replicate, a single adult of either *T. castaneum* or *R. dominica* was placed in the center of the arena and given 2 min to leave the arena. The specific edge as well as the time required to make a decision was recorded for each individual. Beetles leaving the arena on the stimulus edge (e.g., upwind edge nearest to the attractant) were considered to have a positive response to the attractant, while beetles leaving on one of the other three boundaries (non-stimulus edges) of the arena were considered to have a negative response. If no choice was made within the timeframe, the individual was excluded from the final analysis. Beetles in this assay came from one of the density treatments described above. For both species there were 24 replicate individuals evaluated for each treatment combination (species × density × attractant type).

### Release-recapture assay

A release-recapture experiment was conducted for both *T. castaneum* and *R. dominica* using the stimuli listed in Table 1. Assays for *T. castaneum* were conducted in a 4.8 × 2.1 × 6 m L:H:W environmental chamber (Percival Instruments, Perry, IA, USA) set at constant conditions (27.5 °C, 60% RH, and 14:10 L:D) with a layer of craft paper on the floor to aid movement of beetles. Six commercial pitfall traps (Storgard Dome^®^ Traps, Trécé, Inc., Adair, OK, USA) were each baited with one of the six different stimuli (Table 1). The pitfall traps were positioned around the perimeter of the environmental chamber. Trap position was re-randomized in each subsequent round of deployment to avoid positional effects. For each release, 100 mixed sex *T. castaneum* from the 100- and 500-beetle densities above as the high density treatments, or directly from stock colony individuals (effective density 18 beetles/jar) as a less crowded treatment, were settled in a piece of cardboard measuring 8 × 8 cm and released at the center of the environmental chamber at the start of the assay. Traps were collected after a period of 24 h and the number of *T. castaneum* in each trap was recorded.

Based on the lower walking capacity of *Rhyzopertha dominica*^55^, we conducted the release-recapture as above except for the following changes. In each replicate, a total of 20 mixed-sex adults reared at colony density (effective density 18 beetles/jar), 50 beetles/jar, or 100 beetles/jar (as above) were settled on corrugated cardboard (8 × 8 cm) and then placed in one corner of a plastic bin (86.3 × 30.5 × 39.4 cm L:H:W). In the opposite corner, diagonally across from the release point in the bin, a pitfall trap (Dome Trap™, Trécé, Inc., Adair, OK, USA) was deployed. The bins were located in a large (4.8 × 2.1 × 6 m, L:W:H) walk-in environmental chamber under constant conditions (as above). The adults were given 24 h to respond to the lures. In a given block by day, two replicates of each treatment listed above were performed. There was a total of n = 8 replicates for each combination of semiochemical treatment, beetle density, and species.

### Volatile collection and characterization

Headspace samples were collected to characterize potential density-mediated volatiles produced by *T. castaneum* and *R. dominica* infested food (10 g of either whole wheat or flour taken from 0, 50, 100 and 500 densities for *T. castaneum* or 0, 10, 50 and 100 densities for *R. dominica*). Negative controls consisted of empty headspace chambers, and were used to identify and eliminate from analysis any background volatiles in the sampling room. Central air was pushed through an activated charcoal filter to purify the air, and then the flow rate was calibrated with a flow meter (Volatile Collection Systems, Gainsville, FL, USA) at 1 L per min. Four headspace volatile chambers (10.2 × 12.7 cm D:H) were attached to the headspace collection apparatus using Polytetrafluoroethylene (PTFE), chemically inert tubes (5 mm ID)*.* Prior to headspace collection, all *T. castaneum* life stages were sieved out of the flour with first a No. 25 (710 μm) then a No. 70 (212 μm) mesh sieve. For *R. dominica*, all adults were removed from the wheat kernels using a No. 10 (2 mm) mesh sieve. The headspace volatiles were collected over a 3 h period using volatile collection traps (VCT; Volatile Collections Systems, Gainsville, FL, USA). Each VCT consisted of a borosilicate glass tube tapered at one end with a stainless-steel screen, followed by 20 mg of Porapak Q™ adsorbent material to trap volatiles, and held in place with borosilicate glass wool and a PTFE compression seal. The headspace volatiles were extracted from the VCTs using 300 or 150 µl of dichloromethane, with the solvent pushed through by inert N_2_ gas. After extraction, 1 μl of tetradecane (190.5 ng at 99% purity) was added as an internal standard for quantification in each of the labeled vials using a microsyringe (7000 Series Modified Microliter™ Syringe, Hamilton, Reno, NV, USA). Between uses all VCTs were washed with 700 μl of dichloromethane in triplicate. All volatile collection vials were sealed with Teflon tape and stored at − 4 °C until GC–MS analysis. There was a total of n = 4–8 replicates per density and species of beetle.

### Gas chromatography coupled with mass spectrometry

All headspace sample extracts were run on an Agilent 7890B gas chromatograph (GC) equipped with an Agilent Durabond HP-5 column (30 m length, 0.250 mm diameter and 0.25 μm film thickness) with He as the carrier gas at a constant 5 mL/min flow and 39 cm/s velocity, which was coupled with an Agilent 5997B mass spectrometer (MS) single-quadrupole detector. The compounds were injected with 1 μL of each sample under splitless mode into the machine. The program began at 35 °C for 1 min followed by 10 °C/min ramping to 300 °C over 26.5 min, and subsequently held for 4 min at 300 °C. After a solvent delay of 3 min, mass ranges between 50 and 550 atomic mass units were scanned. Preliminary assignment of compounds was obtained by comparing sample spectral data with the NIST 14 library through deconvolution. However, the primary goal was evaluating relative differences in emissions among treatments.

### Statistical analysis

For statistical procedures, R Software was used^56^ and α = 0.05, unless otherwise noted. The glm function from the base R Software, Anova function from the *car* package, and glht function from the *multcomp* package were used for the univariate analyses.

To analyze the data from the wind tunnel assay, a generalized linear model based on a binomial distribution was used for each species. The response variable was the edge of the arena on which the beetles exited (stimulus or non-stimulus edge). The two fixed explanatory variables were the semiochemical treatments (TSO, WG, SPB, contaminated food [CF], uncontaminted food [UCF], and Ctrl) and beetle density (10, 50, 100, or 500 beetles), as well as their interaction. Overdispersion was evaluated and found not to be an issue with the model. Log-likelihood tests based on a χ^2^-distribution were used to calculate significance, and upon a significant result from the model, pairwise χ^2^-tests were used with a Bonferroni correction.

To evaluate whether the trap capture of beetles from different densities was affected by the semiochemical treatments, we analyzed the release-recapture assay for each beetle species using a 2-way ANOVA. Because *T. castaneum* exhibit an exponential decay in responding to semiochemicals by distance, with less than 40% making it to a food patch 16 cm away with airflow present, traps in the release-recapture assay were effectively independent from each other. The total number of adults recaptured by the trap was used as the response variable, while the beetle density (colony-reared, 100-, or 500-beetle density) and the semiochemical treatments (TSO, WG, SPB, CF, UCF, and Ctrl) were employed as fixed, explanatory variables, as well as their interaction. The data conformed to the assumptions of normality and homogeneity of variance, and thus no transformation was required. Upon a significant result from the model, Tukey HSD was used for multiple comparisons among the treatments.

To characterize the presence of headspace volatiles from the extracts, raw peak areas were extracted from the gas chromatograms using Unknowns Analysis (Quantitative Analysis Software, v7.1.524.1, Agilent Technologies, Inc., Santa Clara, CA, USA). Samples were analyzed based on deconvolution of spectra, and only those peaks were included that were at least 0.7% of the height of the tallest peak in order to exclude background noise. A single CSV file with the best hit for each compound and peak area was outputted to a CSV, then analyzed in R Software using the package *uafR* and a new automated protocol to streamline aligning peaks and removed contamination (Stratton et al. 2022). After alignment, background volatiles found in the negative control without grain were discarded from the other samples, since these represent transient background volatiles in the general vicinity of headspace collection but are not informative of differences among the treatments. Pairwise Bray–Curtis dissimilarities were calculated among all headspace samples, and non-metric multi-dimensional scaling (NMDS) was used to visualize the differences in volatile emissions among treatments. A total of n = 1000 permutations were used for the ordination procedure. Stress values for the NMDS procedure were < 0.13, indicating that good interpretation was possible. An analysis of similarity (ANOSIM) was used to determine significant differences for headspace volatiles among levels within density (0, 10, 50, 100, 500) and species (*T. castaneum* or *R. dominica*). A total of n = 1,000 permutations were performed for the test. For all multivariate statistics, the R Package *vegan*^57^ and *ecodist*^58^ was used. The mean volatile emissions for each compound in a treatment were analyzed with a generalized linear model based on a quasi-Poisson distribution to account for overdispersion in the dataset using a call to the function *glm* in R. Explanatory variables included density and species. A likelihood ration test based on a chi-square distribution was performed, with α = 0.05. Multiple comparisons employed Tukey HSD using the function *glht* from the *multcomp* package^59^.

## Results

### Wind tunnel assay

**Figure 2:**
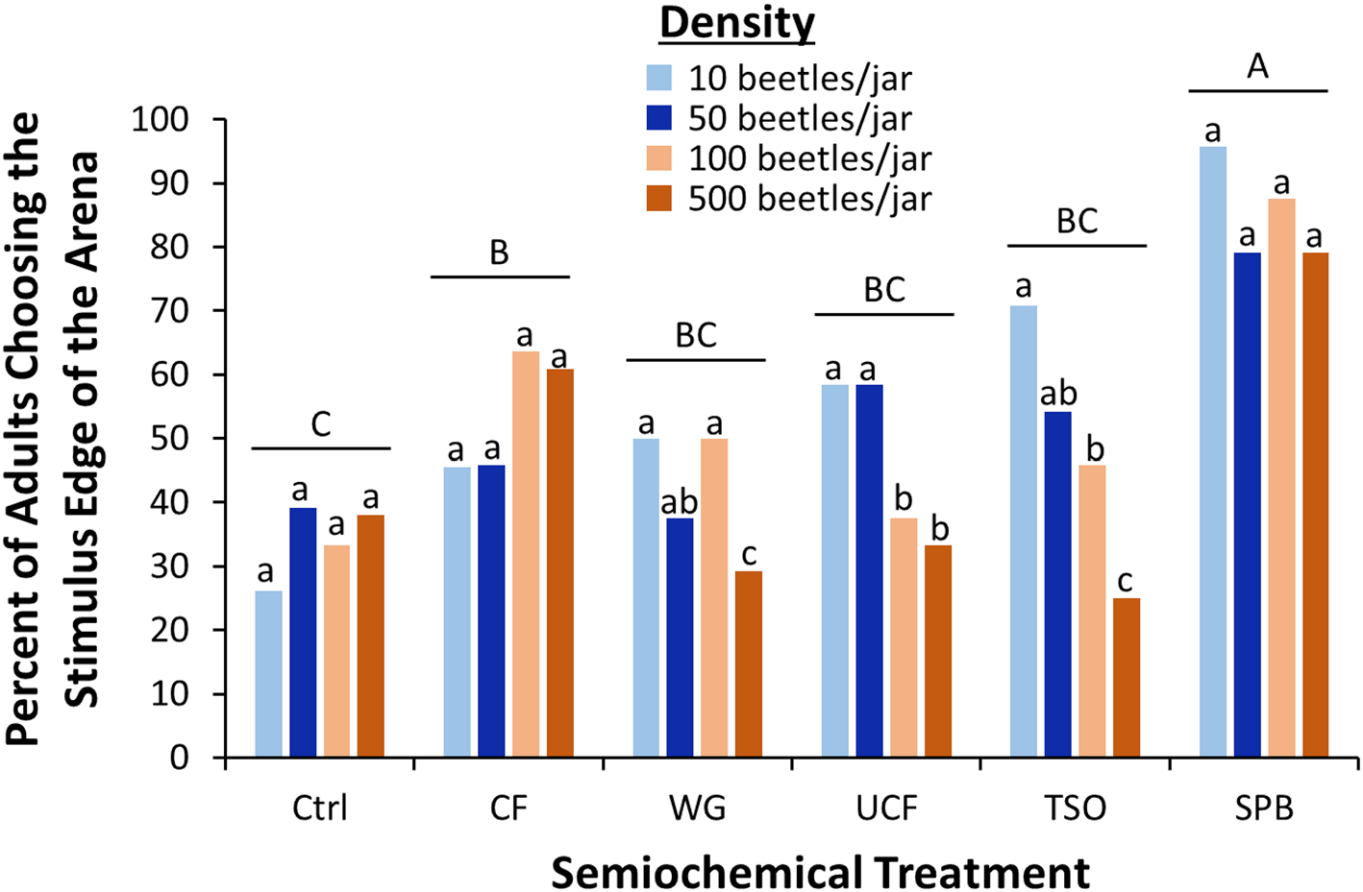
Total percent of *T. castaneum* reared at different densities exiting the release arena in a wind tunnel assay on the upwind stimulus edge for various food and pheromone attractants. Upper case letters represent pairwise comparisons among semiochemical treatments across beetle densities, while lower case letters represent pairwise comparisons among beetle densities within a semiochemical treatment. Bars with shared letters are not significantly different from each other (χ^2^-test, Bonferroni correction). Ctrl unbaited control, CF contaminated food from 500 beetle density, WG wheat germ oil, UCF uncontaminated food with no beetles, *TSO* Trece Storgard Oil, and SPB Stored Product Beetle Pheromone Tab from Insects Limited.

Overall, across treatments only 1.9% of *T. castaneum* did not respond. There were the most nonresponders for the Ctrl (5%) and the CF (5%). There were no nonresponders for any of the other treatments. For *T. castaneum,* density had a significant effect on the number of conspecifics responding positively to stimulus in the wind tunnel assay (*χ*^2^ = 5.70; df = 3; *P* < 0.05; Fig. 2). There was a 13% decreased positive response overall of adults reared at 500-beetle densities compared to those reared at 10-beetle densities. The semiochemical treatment also significantly affected the number of *T. castaneum* adults choosing the stimulus edge (*χ*^2^ = 65.5; df = 5; *P* < 0.0001; Fig. 2). There were 2.5 times more adults leaving on the stimulus edge for the SPB tab compared with the unbaited control, and CF also had a significantly greater positive response compared to the control but less strong than the response to the SPB. All remaining treatments were not significantly different from the controls. The interaction between density and semiochemical treatment was significant (*χ*^2^ = 10.9; df = 5; *P* < 0.05). Importantly, *T. castaneum* attraction to WG, UCF, and TSO (e.g., food semiochemicals) were suppressed by 2–threefold for beetles reared at the 500-beetle density compared to the 10-beetle density, while attraction to the contaminated food (CF) and food and pheromone lure (SPB) remained unchanged over the densities (Fig. 2, pairwise χ^2^-tests).

**Figure 3:**
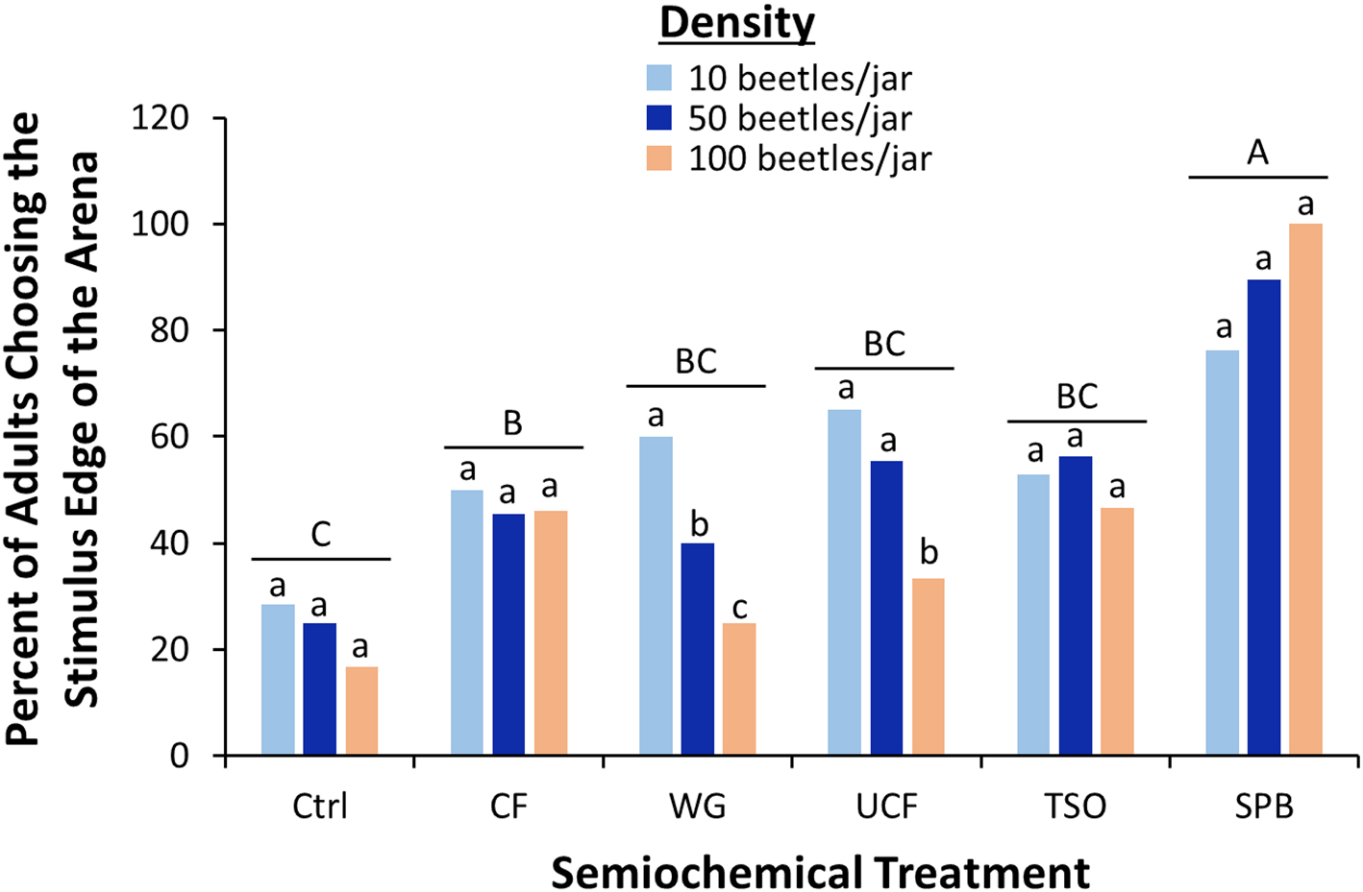
The total percent of *R. dominica* exiting the release arena in a wind tunnel assay on the upwind stimulus edge for various food and pheromone attractants. Upper case letters represent pairwise comparisons among semiochemical treatments across beetle densities, while lower case letters represent pairwise comparisons among beetle densities within a semiochemical treatment. Bars with shared letters are not significantly different from each other (χ^2^-test, Bonferroni correction). Bars with letters omitted are where within semiochemical treatments responses did not differ among beetle densities. *Ctrl* unbaited control, *CF* contaminated food from 500 beetle density, *WG* wheat germ oil, *UCF* uncontaminated food with no beetles, *TSO* Trece Storgard Oil, and *SPB* Stored Product Beetle Pheromone Tab from Insects Limited.

Overall, across treatments a total of 33% of *R. dominica* did not respond, which is typical for this species. There were the least number of nonresponders for the SPB lure (20%) and the most for the CF (47%). Generally, the density at which *R. dominica* were reared did not affect the number leaving on the stimulus edge of the arena (*χ*^2^ = 0.36; df = 3; *P* = 0.55). However, the semiochemical treatment significantly affected attraction of *R. dominica* (*χ*^2^ = 48.0; df = 5; *P* < 0.0001), with 3 times more adults exiting towards the SPB lure than the unbaited control (Fig. 3). The contaminated food was also significantly more attractive than the control, but significantly less than SPB, with all other treatments not being different from the control response. Finally, the interaction between the two variables was not significant (*χ*^2^ = 4.03; df = 15; *P* = 0.55).

### Release-recapture assay

**Figure 4:**
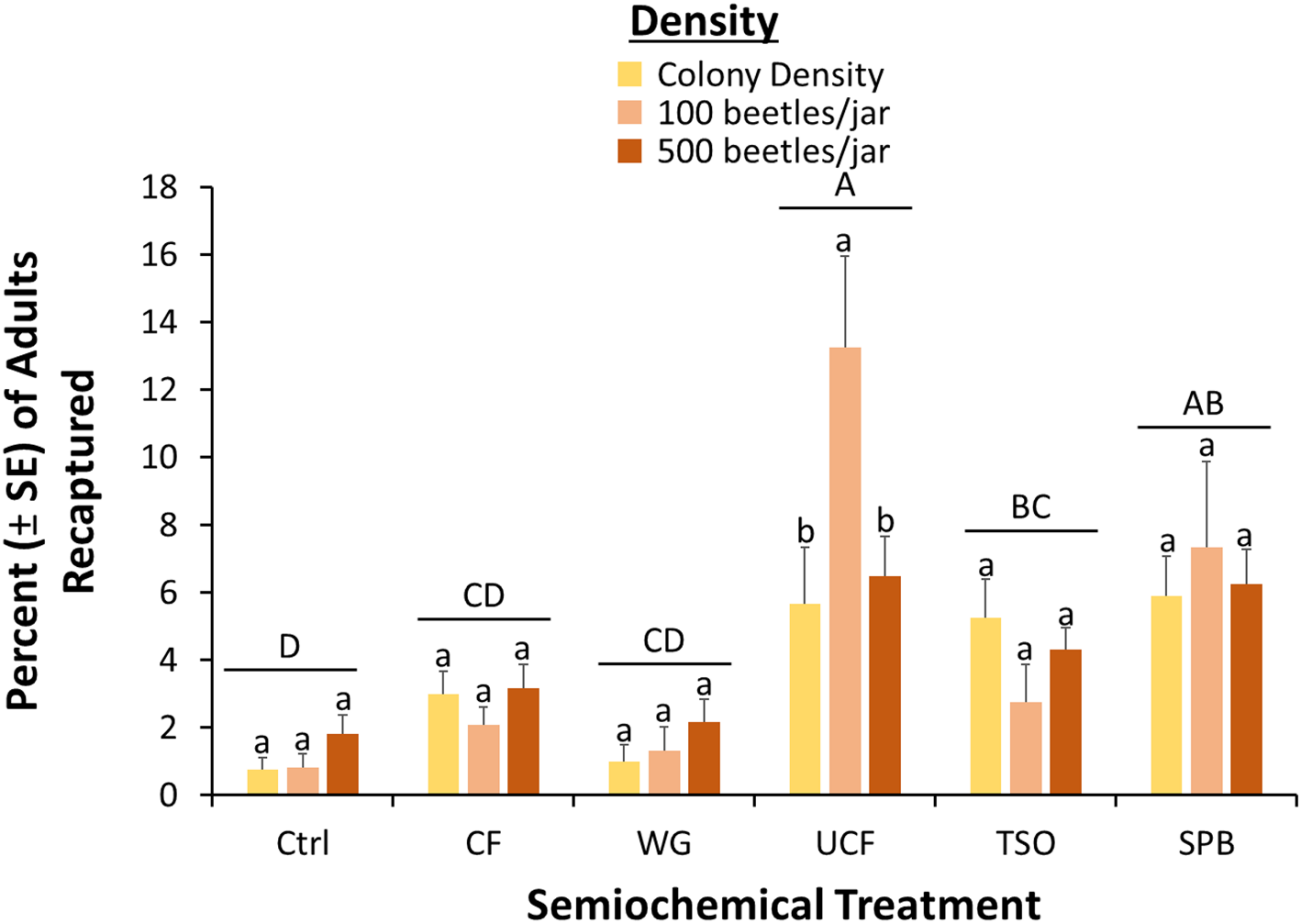
Percent (± SE) of *T. castaneum* adults reared at different densities and recaptured in pitfall traps baited with different semiochemical treatments under constant 27.5ºC and 60% RH after 24 h. A total of N = 8 replicates for each treatment combination. Upper case letters represent comparisons among semiochemical treatments across densities, while lower case letters represent comparisons within a semiochemical treatment among densities. Bars with shared letters are not significantly different from each other (Tukey HSD, α = 0.05). *Ctrl* unbaited control, *CF* contaminated food from 500 beetle density, *UCF* uncontaminated food with no beetles, *TSO* Trece Storgard Oil, and *SPB* Insects Limited SPB lure.

We recaptured 46% of the *T. castaneum* beetles that were released, suggesting that valid interpretation is possible. Density did not significantly affect the capture of *T. castaneum* adults in the overall model (*χ*^2^ = 1.50; df = 2; *P* = 0.47; Fig. 4), but for UCF the intermediate density had significantly greater captures than the colony density or the 500 beetles/jar density. Average captures ranged from 3.5 and 4.2 beetles per trap when reared at stock colony levels and the 100 beetles/jar density, respectively. However, the semiochemical treatments in traps significantly altered the capture of adults (*χ*^2^ = 118; df = 6; *P* < 0.0001). In particular, traps with the SPB tab and the uncontaminated food (UCF) captured 5.7 and 7.4 times more adults, respectively, compared to unbaited control traps. In addition, the interaction between density and the semiochemical treatment was significant (*χ*^2^ = 21.5; df = 12; *P* < 0.05), with greater recapture of adults reared at 100 adults/jar in traps baited with uncontaminated food compared to the other densities, but no significant differences among densities for traps baited with other stimuli.

**Figure 5:**
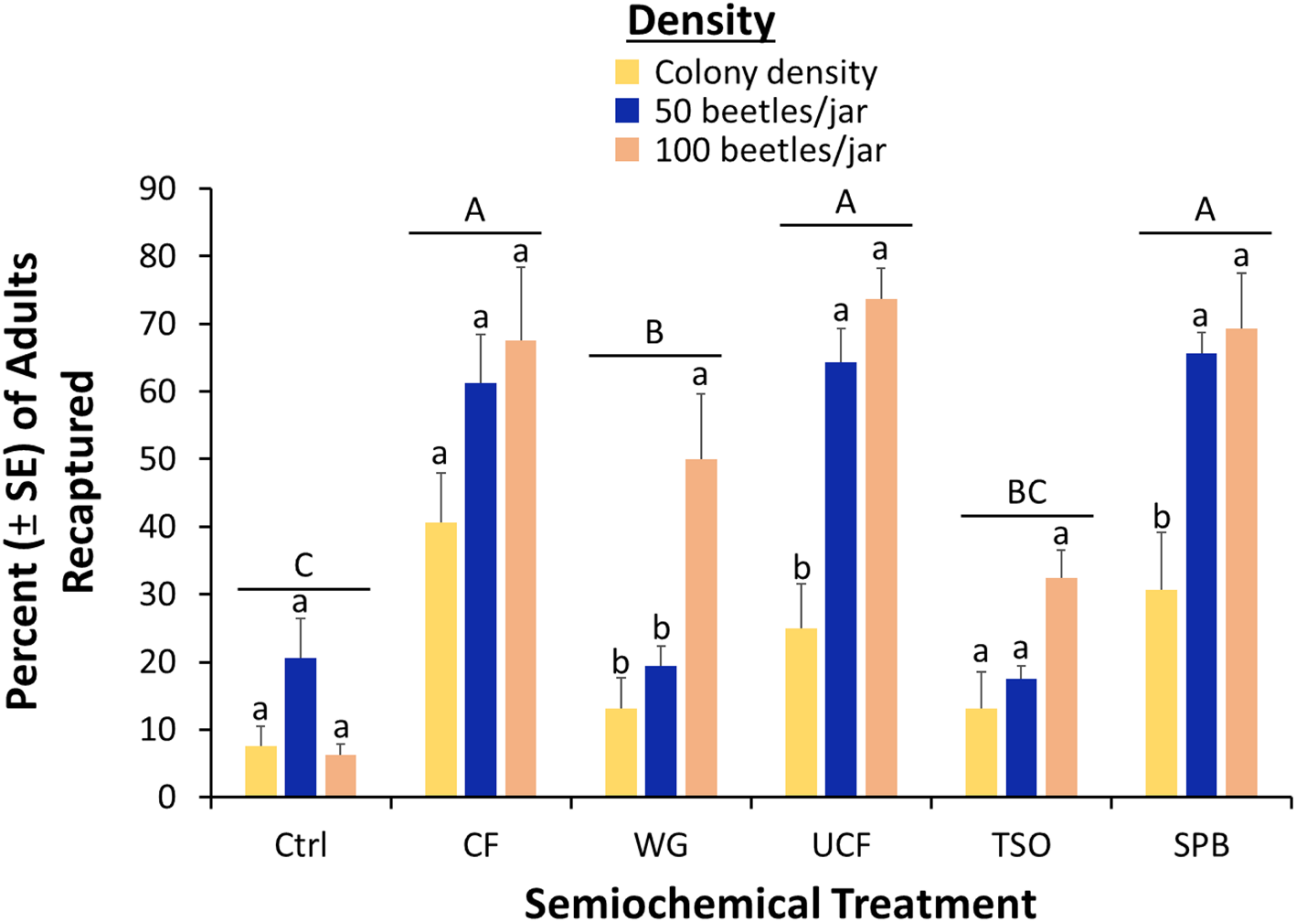
Percent (± SE) of *R. dominica* adults reared at different densities and recaptured in pitfall traps baited with different semiochemical treatments under constant 27.5ºC and 60% RH after 24 h. A total of N = 8 replicates for each treatment combination. Upper case letters represent comparisons among semiochemical treatments across densities, while lower case letters represent comparisons within a semiochemical treatment among densities. Bars with shared letters are not significantly different from each other (Tukey HSD, α = 0.05). Colony density was effectively 18 beetles/jar. *Ctrl* unbaited control, *CF* contaminated food from 100 beetle density, *UCF* uncontaminated food with no beetles, *TSO* Tréce Storgard oil, and *SPB* Insects Limited SPB lure.

Similar to *T. castaneum*, we recaptured 38% of the *R. dominica* that were released, suggesting valid interpretation of the data was possible. Overall, density significantly affected the recapture of *R. dominica* in traps (*χ*^2^ = 64.1; df = 2; *P* < 0.0001; Fig. 5), with only about half as many adults captured in traps with colony densities compared to 50 and 100 adults/jar. The semiochemical treatment significantly affected recapture of *R. dominica* as well (*χ*^2^ = 157 df = 6; *P* < 0.0001), with traps baited with CF, SPB, or UCF capturing 4.7–4.9-fold more adults than unbaited controls. There was a significant interaction between density and semiochemical treatment interaction on trap captures of *R. dominica* (*χ*^2^ = 24.9, df = 12; *P* < 0.01), with 50 and 100 densities showing 2.1–3.8-fold higher increased response to the SPB lure, UCF, and WG relative to the lower colony density. Density did not affect response to the other semiochemical treatments.

### Volatile characterization

**Figure 6:**
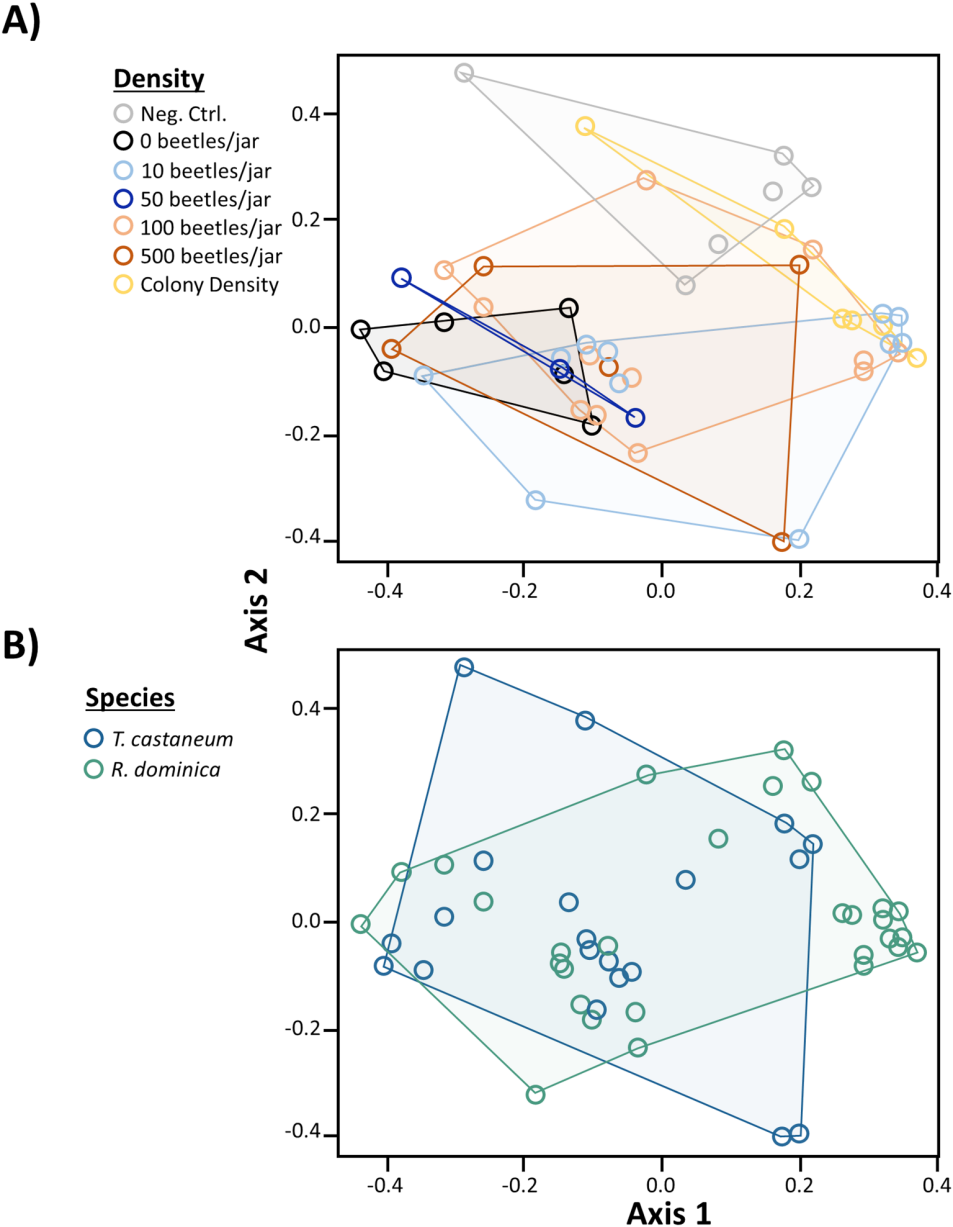
Ordination (non-metric multidimensional scaling plot) of density emissions from wheat based on Bray–Curtis indices calculated in a pairwise fashion among *R. dominica* and *T. castaneum* A) reared at different densities (0—black or grey, 10—dark blue, 50—light blue, 100—medium blue, 500—green, colony density—purple) after 3–4 weeks, and B) differences in volatiles by species-specific emissions with *R. dominica* in green and *T. castaneum* in blue. Stress was less than 0.07, suggesting valid interpretation is possible. There were a total of n = 5–12 replicates per density, and n = 20–29 replicates per species.

**Figure 7:**
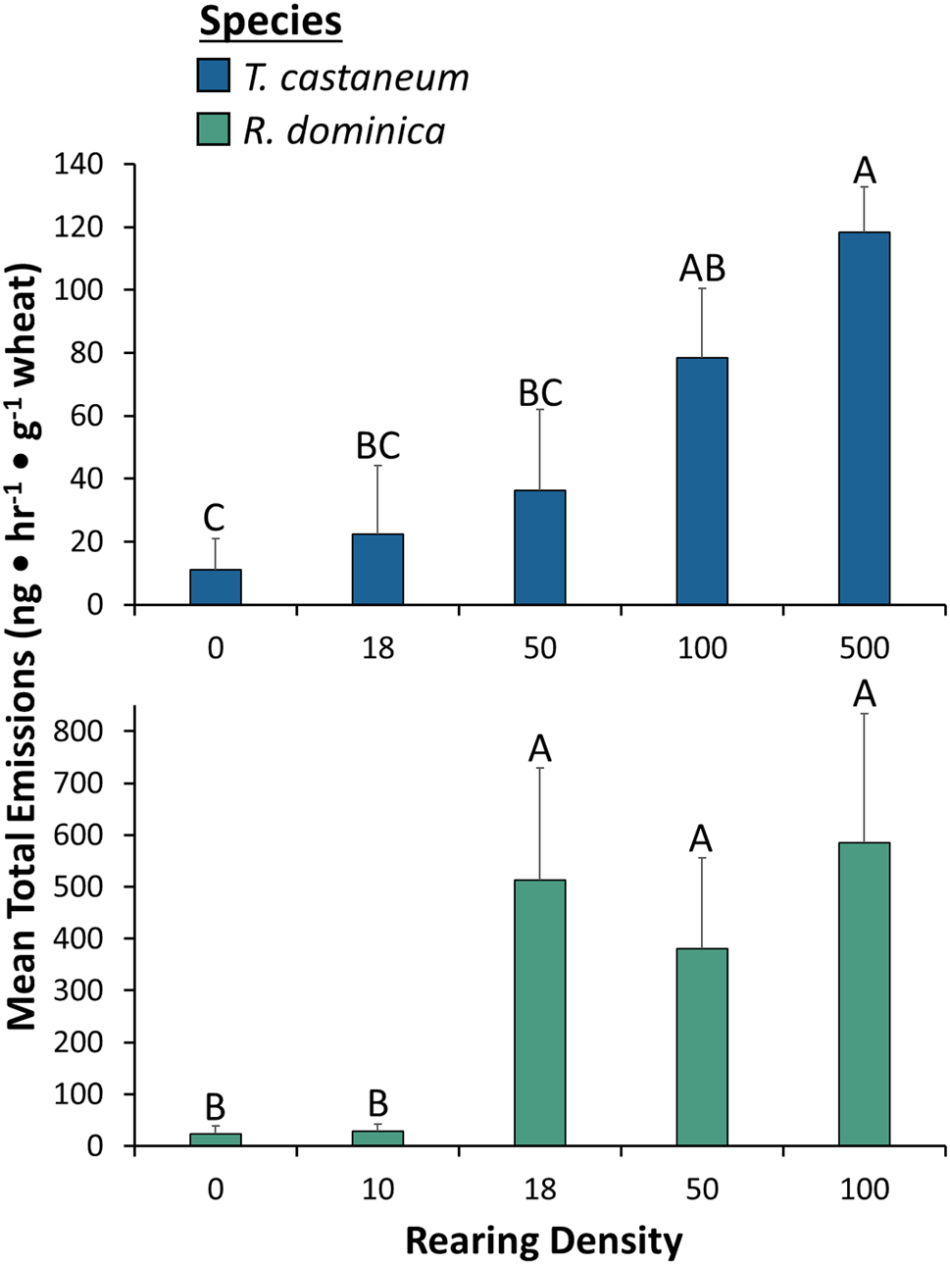
Mean total volatile density emissions by *T. castaneum* (top panel) and *R. dominica* (bottom panel) after 4 weeks based on density. Bars with shared letters are not significantly different from each other (Tukey HSD, α = 0.05). To be included, compounds were required to be at least 0.7% the height of the major peak in the sample.

In total, we recorded the presence of 55 chemical compounds from whole grain or flour samples taken from containers containing different densities of *T. castanuem* and *R. dominica* (Table 2). Volatile emissions from the different densities of *R. dominica* contained 5–24 fewer compounds on average, compared to the treatment with wheat only (Table 2). Additionally, we found that as the density increased, so too did the abundance of most of the compounds. Importantly, there were significant density-dependent differences in the volatile composition of headspace emitted from the grain by *R. dominica* and *T. castaneum* (ANOSIM: R = 0.131, P < 0.01; Fig. 6A). The compounds most associated with the high density treatments of *R. dominica* (e.g., 50 and 100) were dodecanal, 2-pentyl ester cyclopentanecarboxylic acid, and 4-methylpentyl ester-2-thiophenecarboxylic acid (Table 2). By contrast, toluene, propyne, and heptadecane, were present in all densities of *T. castaneum*, but highest in the 100 beetle density. In addition, the 100 beetle density of *T. castaneum* was enriched in more compounds such as octane, dodecane, and nonanal, compared to the other densities. Moreover, there were significant species-specific differences in the emissions of volatiles (ANOSIM: R = 0.085, P < 0.02; Fig. 6B). Toluene, propyne, heptadecane, and (*Z*,*Z*,*Z*,*Z)*-1,5,9,9-tetramethyl-1,4,7-cycloundecatriene were most characteristic of *T. castaneum*, while dodecanal, 2-pentyl ester cyclopentanecarboxylic acid, and 4-methylpentyl ester-2-thiophenecarboxylic acid, and nonadecane were most dominant in *R. dominica*. Total volatile emissions were significantly affected by density (*χ*^2^ = 21.0; df = 5; *P* < 0.001; Fig. 7). There were 3-, 12-, 11-, and 11-fold higher volatile emissions in colonies with 10, 50, 100 beetles, and colony density compared to no beetles (Fig. 7). The species also significantly affected the total emissions from grain (*χ*^2^ = 18.8; df = 1; *P* < 0.0001; Fig. 7), but not its interaction with density (*χ*^2^ = 0.801; df = 3; *P* = 0.85). In particular, *Rhyzopertha dominica* colonies had about eightfold higher emissions than *T. castaneum* colonies.

## Discussion

In this study, we examined the behavioral response of the stored product pests, *T. castaneum* and *R. dominica,* to food and pheromonal cues after exposure to varying densities. *Tribolium castaneum* held at certain higher population densities were less likely to respond positively to food cues in comparison to those reared at lower densities when pheromone was absent. For example, *T. castaneum* conspecific responses to contaminated flour or the lure that had food and pheromone was not changed. In fact, we found that the contaminated flour contained the pheromone from *T. castaneum,* supporting this point. This suggests that response to pheromones remained unchanged. By contrast, *R. dominica* reared at higher densities showed higher sensitivity to food and pheromone cues, exhibiting increased response compared to lower densities in the release-recapture study, though not in the wind tunnel. Interestingly, some unique compounds were emitted from high density treatments by each species. For example, nonadecane and pentanoic acid ester were unique to *R. dominica*, while 1-tridecene and (1*E*)-3,7,11-trimethyl-1,6,10-dodecatrien-3-ol were unique to *T. castaneum*. Overall, there seemed to be species-specific responses to high density. Headspace from *T. castaneum* was dominated by toluene, propyne, heptadecane, and (*Z*,*Z*,*Z*,*Z*)-1,5,9,9-tetramethyl-1,4,7-cycloundecatriene, while dodecanal, 2-pentyl ester cyclopentanecarboxylic acid, and 4-methylpentyl ester-2-thiophenecarboxylic acid, and nonadecane comprised *R. dominica* headspace.

Here, we have considered how exposure to density-mediated cues affects stored product beetle behavior. These density-mediated cues may have included volatiles from oviposited eggs, excreted frass, deposited trace cuticular hydrocarbons and glandular secretions from tunneling by adults, and a food source imbued with a variety of volatile compounds, including aggregation pheromones and stress-related compounds such as methyl- and benzoquinones in the case of *T. castaneum*^14,60^. For the volatile collection treatments in our study, we used increasingly small sieves to remove beetles, larvae, and eggs. However, frass and residual volatiles in the grain (including density-related compounds) likely remained. For example, 1-tridecene has previously been collected from headspace of *T. castaneum*, and assumed to be a sex pheromone based on homology to the same compound from another tenebrionid^61^. There were also trace amounts of 1-pentadecene, which has been described as a larval frass volatile from *Tribolium* spp^62^. Thus, when we observed behavioral effects or volatile differences among different densities of beetles, we are primarily describing differential responses to these density-mediated cues. Prior to this research, the effect of density on response to conspecific aggregation pheromones for *T. castaneum*, but not food cues, was investigated^14^. In that study, researchers found that *T. castaneum* reared at 50 or 250 adults on 10 g of flour chose a clean grain volatile source 10- to 6-times more often than a grain volatile source from 1250 adult *T. castaneum*.

In the current study, we also found that *T. castaneum* was the most susceptible to modulation of its foraging behavior by density in the wind tunnel, but not in the release-recapture experiment. By contrast to *T. castaneum*, we observed that *R. dominica* was more affected in the release-recapture experiment than the wind tunnel assay to density-mediated cues at least under the levels evaluated here. It may be that such a pattern arises as a result of the difference in the life histories of our two species. Since *R. dominica* is a primary pest, it is less likely to be exposed to crowded conditions in environments that consist of many tonnes of commodities being stored for variable, sometimes short periods of time. In fact, we observed that *R. dominica* from high density rearings was captured more often in traps baited with cues that may also have pheromone. Over the range of densities tested, the total emissions by *R. dominica* plateaus by 50 individuals/jar, and remains steady at 100 individuals/jar and for the colony density (see Fig. 6). Interestingly, *T. castaneum* appeared somewhat more attracted to grain when contaminated by conspecifics compared to the negative control, though this effect was moderate, because their response to clean grain was not significantly different from the negative control or contaminated food. Indeed, we found trace amounts of pheromone in clean grain from headspace, which may explain why no differences were observed in our study. As an alternate hypothesis, it may be possible that the behavioral response of *R. dominica* to food semiochemicals was not affected, because they may only be responsive to host volatiles during foraging. For example, *R. dominica* are recognized as weak walkers and may locate food sources by chance when they are walking. Cordeiro et al.^63^ showed that *R. dominica* foraging in wheat tended to revisit the same areas where they had previously fed. In addition, those authors found there was evidence that *R. dominica* use cues associated with their feeding while in a grain mass. Finally, that study also showed incorporating fine materials from high infestation level grain by conspecifics at different ratios affected movement by *R. dominica* in wheat. By contrast, some studies found *R. dominica* may only respond to semiochemical cues when locating areas to land during flight from a distance, because of their strong dispersal flight capacity^30^. Future studies should employ a tethered flight mill^64–67^ to determine whether flight capacity in the presence of food cues by *R. dominica* is affected by density. Additionally, the conditions that trigger dispersal and the specific volatiles they may be using for orientation as they find routes to infest facilities are all open questions that should be addressed.

Both *T. castaneum* and *R. dominica* have evolved a complex system of glandular and volatile emissions, as well as odor-reception for dealing with crowded situations^10,68^. In support of this, we found the density volatile bouquets for both *T. castaneum* and *R. dominica*, were species-specific, and vary with the density of beetles. In particular, we found increasing total emissions of volatiles compared with the control with increasing density for both *T. castaneum* and *R. dominica*. Generally, we found that as beetle density increased, the diversity of compounds decreased for *R. dominica* but not *T. castaneum*. It is likely that increased densities of insects supported more microbial contamination, which may also be involved in emissions^42^. In fact, we observed octane, 2-methyl-1-butanol, and hexanal enriched at the high density treatments of the species, which are all considered to be microbial volatiles^69^.

In prior work, the amount of volatiles produced by *T. castaneum* adults increased with increasing insect density, including for methyl-1,4-benzoquinone, ethyl-1,4-benzoquinone, and 1-tridecene^61,70^. For example, Duehl et al.^14^ tested a range of *T. castaneum* densities from near zero to over 1200, and found pheromone production (4,8-dimethyldecanal) peaked at 500 conspecifics, which produced about 200 ng/h, and methyl and ethyl 1,4-benzoquinone,which can act as repellents, peaked above 1200 individuals, producing about 600 ng/h. In contrast to our study, *T. castaneum* from this previous was found not prefer cues from high densities cultures of 250–1250 conspecifics when reared at 50–1250 individuals per 10 g flour^14^. However, we reared beetles on double the amount of flour (20 g) as prior work (10 g), leading to absolute beetle densities that were effectively halved by comparison. This may mean that there was a lower concentration of density cues that would likely act as repellents (e.g., benzoquinones) in the current study than in the prior one, which may be why repellency was not observed in our study.

Both *R. dominica* and *T. castaneum* produce pheromone when feeding on a high quality food source. *Rhyzopertha dominica* is strongly attracted to infested wheat when its pheromone is present, regardless of other factors. or volatiles^71^. In our study, *R. dominca* were still attracted to wheat even when high levels of volatiles associated with feces were present. For example, fenchone was associated with *R. dominica*-infested wheat, while 1-tetradecanol and methyl-decanoate were associated with *R. dominica* feces. However, response to infested grain in high density situations seems to have a disproportionate effect on the foraging of *T. castaneum*. Generally, *T. castaneum* or most species with an aggregation pheromone may be expected to exhibit a positive response to the food resource and increase up to a certain density, then subsequently to show decreased response as density continues to increase. In addition, in prior work, researchers found that the production of 4,8-dimethyldecanal was weakly correlated with the production of methyl and ethyl benzoquinones^16^. In our study, we found that the behavioral response by *T. castaneum* to food cues decreased after exposure to high densities, but notable exceptions were where pheromone or both pheromone and food were present. It is possible that exposure to repellents (e.g., quinones) from the high-density treatment could be responsible for the lower response to food in beetles from the high density treatment, but perhaps the pheromone can override that behavioral response. This latter aspect merits further research to disentangle specific mechanisms, identify if there is an eliciting compound for this behavior, and whether it can be used for behaviorally-based management of *T. castaneum*. In addition to behavioral mechanisms, there may be physiological processes at play in exposure to different densities, which may cause cascades internally in metabolites and gene regulation. Future work should evaluate how density may alter the homeostasis of these internal mechanisms. Finally, future work should explore 1) whether there is a robust response to the compounds by beetles via gas chromatography coupled with electroantennographic detection (GC-EAD), and 2) for promising volatiles identified from GC-EAD, culture the beetles with these compounds to assess whether the compounds affect beetles’ behavioral responses to food cues.

While the current study was primarily focused on the effect of intraspecific density cues on conspecific response to food and pheromone cues, it may be worthwhile to determine whether density cues from *R. dominica*, for instance, affect the behavioral response of *T. castaneum* and vice versa, and whether they can in fact perceive them. Under typical grain storage conditions, multiple species will often be present in a single area, composing a unique ecological community adapted to utilizing anthropogenic granaries. In the vast majority of cases, there will be multiple primary and secondary pests attacking grain at a food facility^72,73^, and it may be worthwhile to understand how crowding cues from one species affects another species. For example, perhaps the density-mediated cues from *T. castaneum* have a similarly downregulating effect on other species, and thus may be of broader interest than if they were specific only to *T. castaneum*.

Currently, behaviorally-based management approaches are being developed for stored product insects^74^. For the first time, we have shown that crowding may affect the attractiveness of food cues to *T. castaneum*, and thus may affect the beetles’ ability to locate new host food patches. However, when common commercial lures were deployed in traps, they did not affect capture of *T. castaneum* or *R. dominica*. Thus, food facilities experiencing different densities of infestation may experience relatively stable trap performance in long-term monitoring programs. Nonetheless, there are some worthwhile follow-up studies to be performed at the intersection of behaviorally-based management and how density affects performance. For example, it would be useful to understand whether exposure to high concentrations of benzoquinones or other isolated density cues impact orientation to traps. The stimuli in this study may be most useful as a repellent, but in high concentrations it would be interesting to understand if it overrides orientation to pheromone with behaviorally-based tools. Follow-up studies could then assess whether briefer bouts of exposure result in the same behavioral changes to pheromones. Overall, this study presents an interesting step forward in understanding the basic ecological relationships between stored product insects, density, and the repercussions that this has on host-finding behavior. Future work will be well-positioned to further elucidate these relationships and determine whether any of the volatiles tentatively identified in this study may be useful from an integrated pest management perspective.

### Supplementary Information

Supplementary Information 1.

Supplementary Information 2.

## Data Availability

Ponce, Marco A.; Ranabhat, Sabita; Bruce, Alexander; Van Winkle, Taylor; Campbell, James F.; Morrison, William R. (2024). Data from: Density-mediated emissions by *Rhyzopertha dominica* (Coleoptera: Bostrichidae) and *Tribolium castaneum* (Coleoptera: Tenebrionidae) modulates foraging by conspecifics. Ag Data Commons. Dataset. 10.15482/USDA.ADC/24851604.v1. Accessed 2024-05-21.
