# Supplementary material for: Density-mediated foraging behavioral responses of Rhyzopertha dominica (Coleoptera: Bostrichidae) and Tribolium castaneum (Coleoptera: Tenebrionidae)
Source: Sci Rep. 2024 May 28;14:12259. doi: 10.1038/s41598-024-62277-8 (PMC11133452; doi:10.1038/s41598-024-62277-8)
Supplement: Supplementary file 1 — Supplementary Information 1. [file 41598_2024_62277_MOESM1_ESM.pptx]

## Slide 1
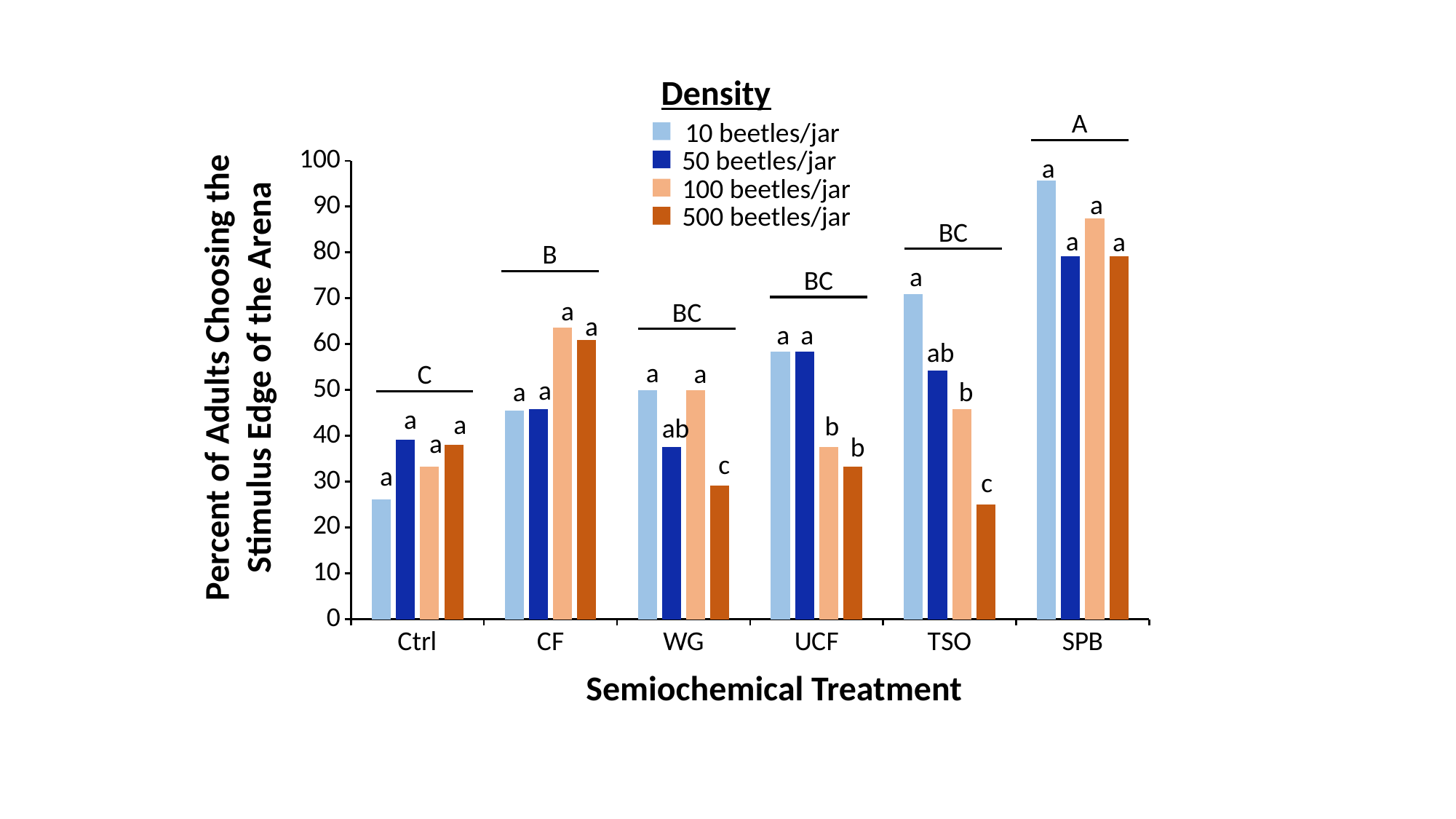

Density
A
10 beetles/jar
50 beetles/jar
100 beetles/jar
500 beetles/jar
### Chart
| Category | | | | |
|---|---|---|---|---|
| Ctrl | 26.08695652173913 | 39.130434782608695 | 33.33333333333333 | 38.095238095238095 |
| CF | 45.45454545454545 | 45.83333333333333 | 63.63636363636363 | 60.86956521739131 |
| WG | 50.0 | 37.5 | 50.0 | 29.166666666666668 |
| UCF | 58.333333333333336 | 58.333333333333336 | 37.5 | 33.33333333333333 |
| TSO | 70.83333333333334 | 54.166666666666664 | 45.83333333333333 | 25.0 |
| SPB | 95.65217391304348 | 79.16666666666666 | 87.5 | 79.16666666666666 |a
a
BC
a
a
B
a
BC
a
BC
a
a
a
ab
Percent of Adults Choosing the Stimulus Edge of the Arena
a
a
C
a
a
b
a
a
b
ab
a
b
c
a
c
Semiochemical Treatment

## Slide 2
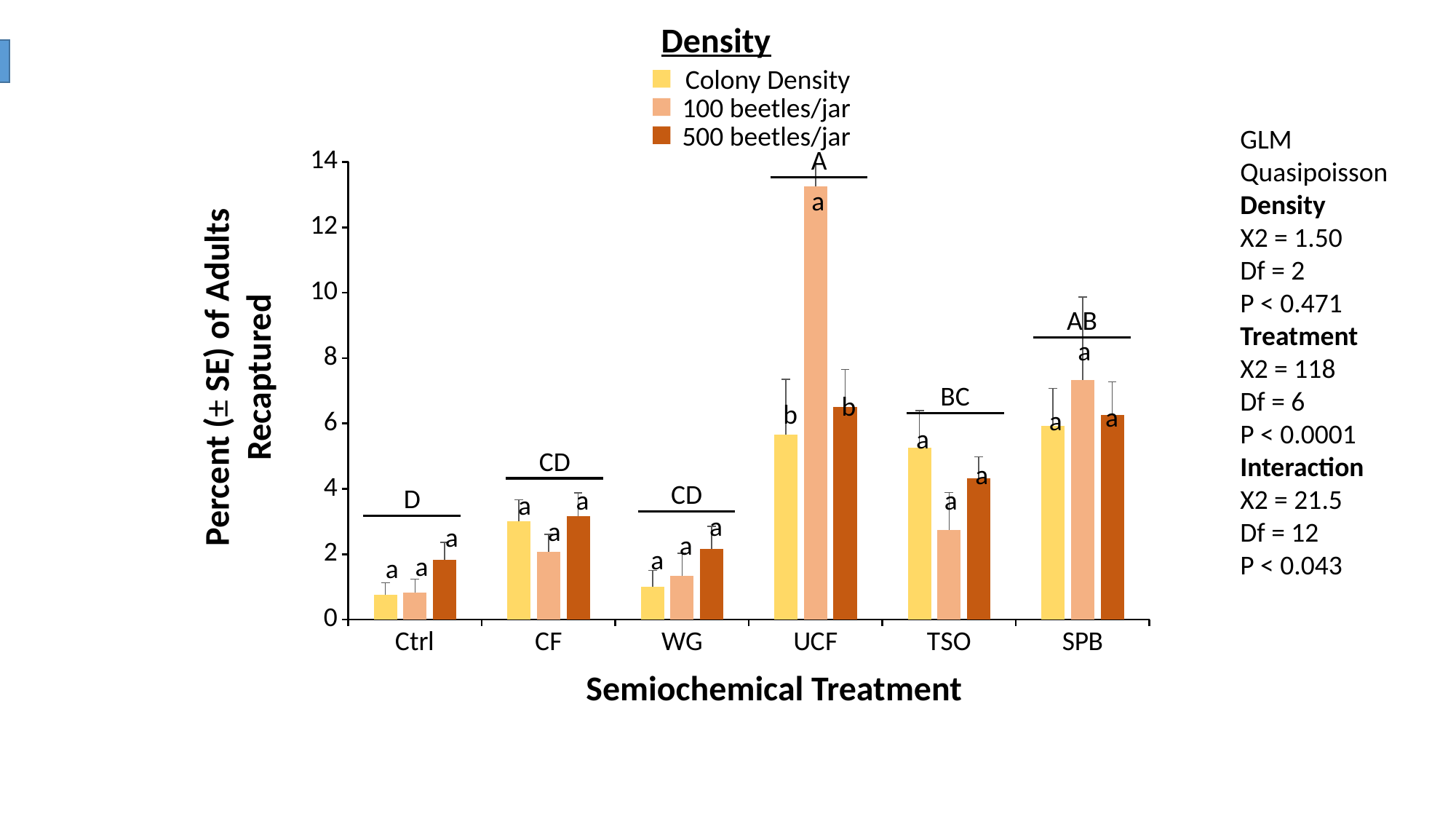

Density
Colony Density
100 beetles/jar
500 beetles/jar
A
### Chart
| Category | | | |
|---|---|---|---|
| | 0.75 | 0.8333333333333334 | 1.8333333333333333 |
| | 3.0 | 2.0833333333333335 | 3.1666666666666665 |
| | 1.0 | 1.3333333333333333 | 2.1666666666666665 |
| | 5.666666666666667 | 13.25 | 6.5 |
| | 5.25 | 2.75 | 4.333333333333333 |
| | 5.916666666666667 | 7.333333333333333 | 6.25 |a
AB
a
Percent (± SE) of Adults Recaptured
BC
b
b
a
a
a
CD
a
CD
D
a
a
a
a
a
a
a
a
a
a
Semiochemical Treatment
GLM
Quasipoisson
Density
X2 = 1.50
Df = 2
P < 0.471
Treatment
X2 = 118
Df = 6
P < 0.0001
Interaction
X2 = 21.5
Df = 12
P < 0.043

## Slide 3
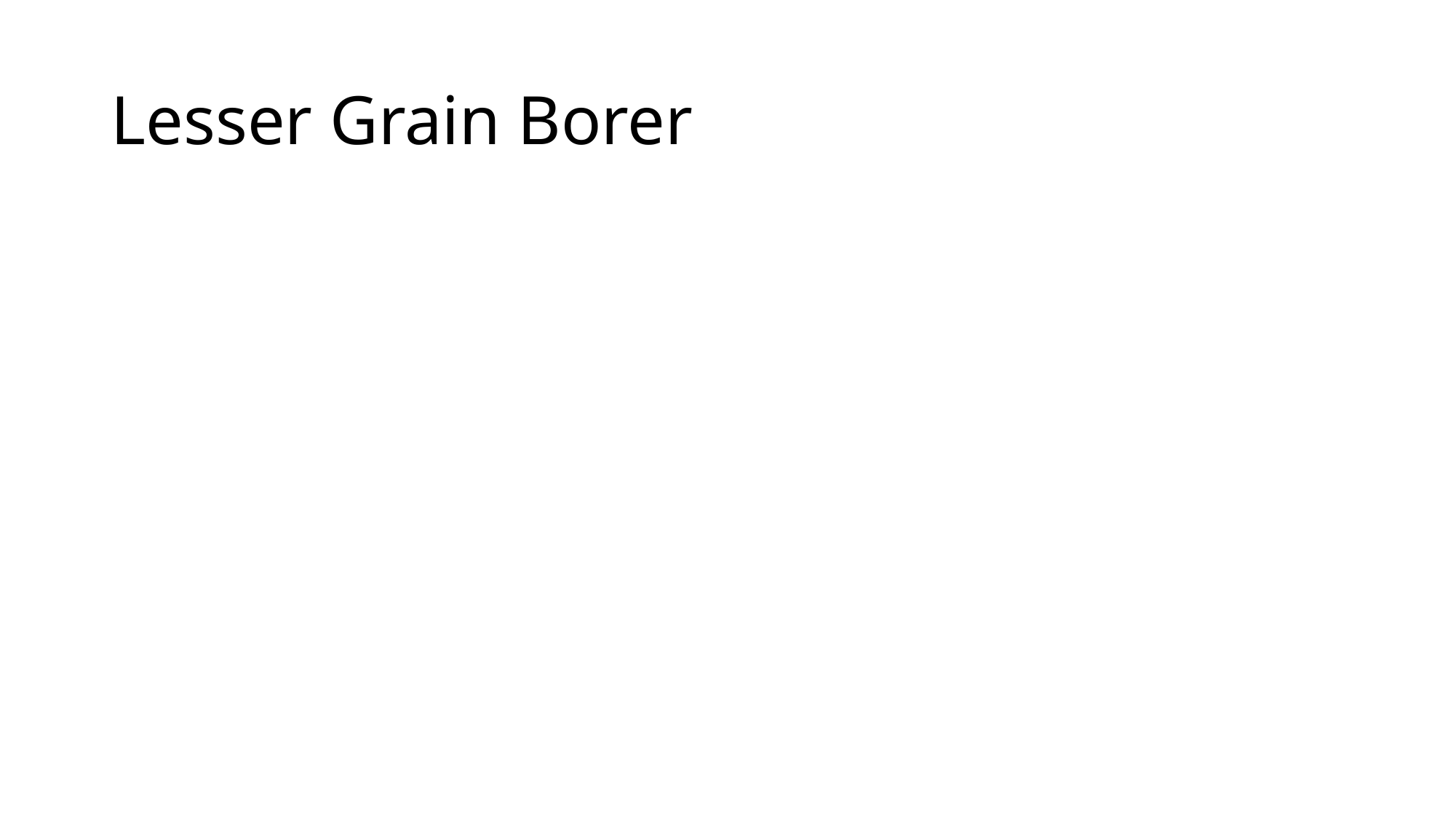

# Lesser Grain Borer

## Slide 4
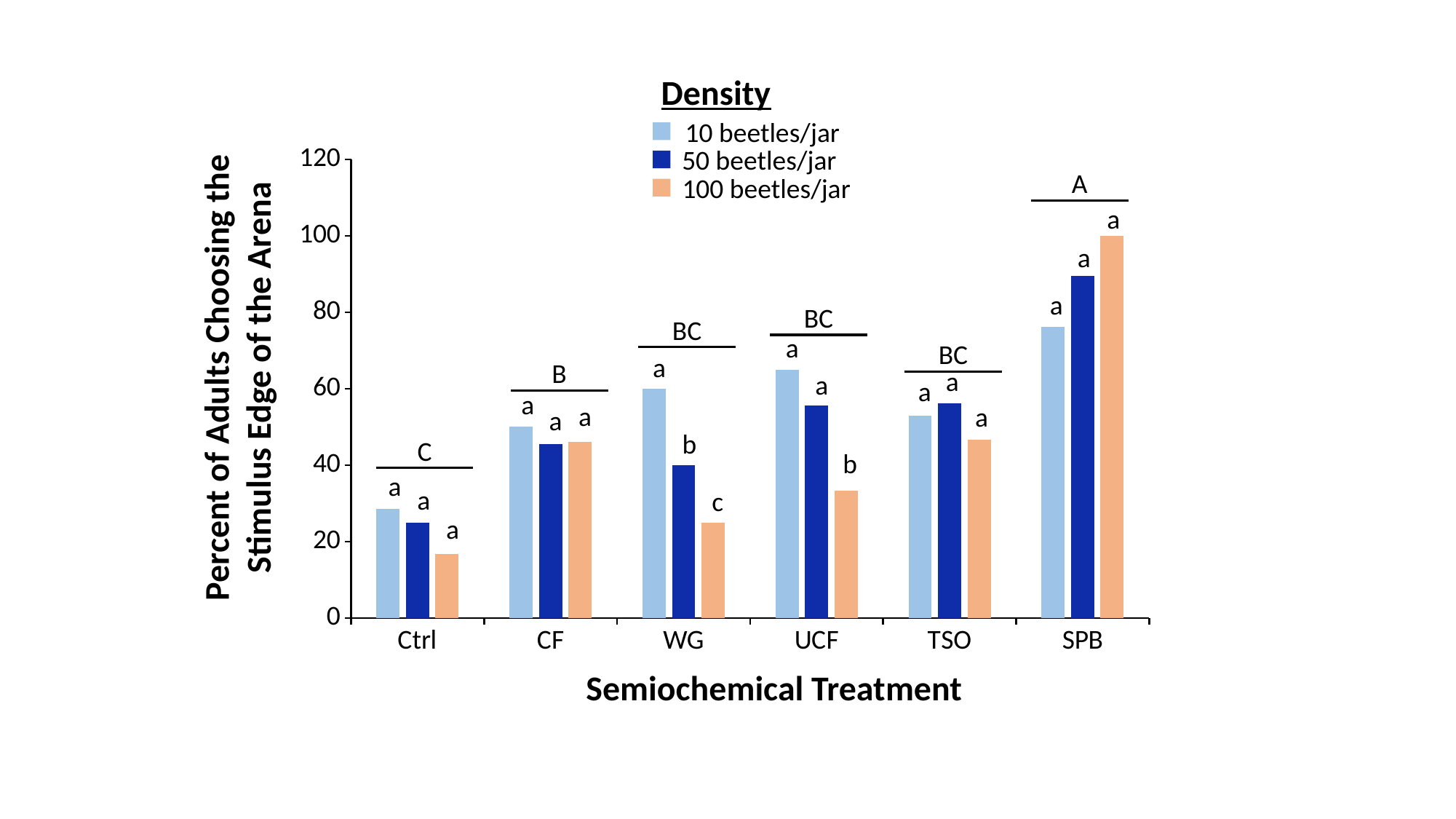

Density
10 beetles/jar
50 beetles/jar
100 beetles/jar
### Chart
| Category | | | |
|---|---|---|---|
| Ctrl | 28.57142857142857 | 25.0 | 16.666666666666664 |
| CF | 50.0 | 45.45454545454545 | 46.15384615384615 |
| WG | 60.0 | 40.0 | 25.0 |
| UCF | 65.0 | 55.55555555555556 | 33.33333333333333 |
| TSO | 52.94117647058824 | 56.25 | 46.666666666666664 |
| SPB | 76.19047619047619 | 89.47368421052632 | 100.0 |A
a
a
a
BC
BC
a
Percent of Adults Choosing the Stimulus Edge of the Arena
BC
a
B
a
a
a
a
a
a
a
b
C
b
a
a
c
a
Semiochemical Treatment

## Slide 5
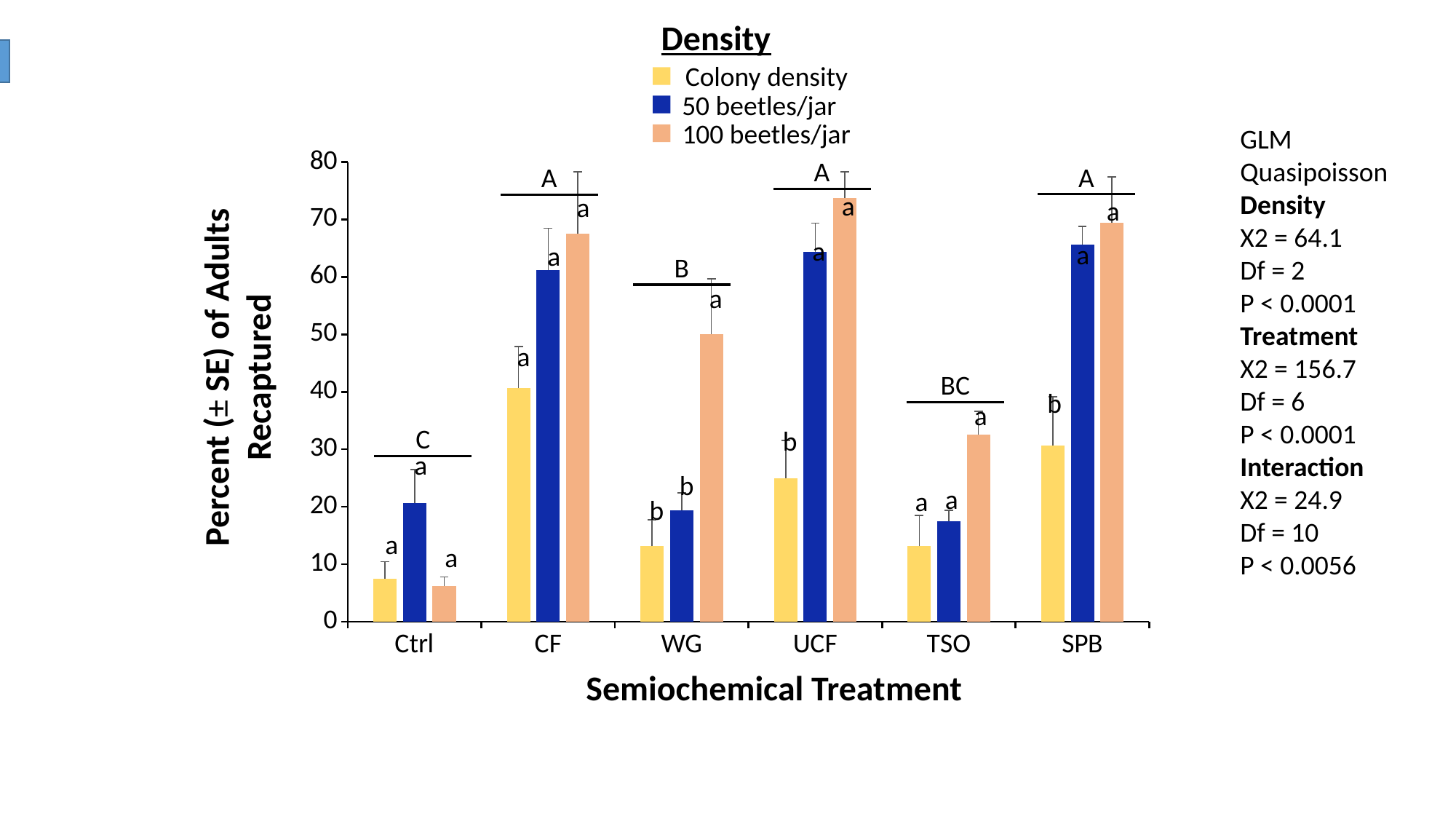

Density
Colony density
50 beetles/jar
100 beetles/jar
### Chart
| Category | 7.5 | | |
|---|---|---|---|
| Ctrl | 7.5 | 20.625 | 6.25 |
| CF | 40.625 | 61.25 | 67.5 |
| WG | 13.125 | 19.375 | 50.0 |
| UCF | 25.0 | 64.375 | 73.75 |
| TSO | 13.125 | 17.5 | 32.5 |
| SPB | 30.625 | 65.625 | 69.375 |A
A
A
a
a
a
a
a
a
B
a
Percent (± SE) of Adults Recaptured
a
BC
b
a
C
b
a
b
a
a
b
a
a
Semiochemical Treatment
GLM
Quasipoisson
Density
X2 = 64.1
Df = 2
P < 0.0001
Treatment
X2 = 156.7
Df = 6
P < 0.0001
Interaction
X2 = 24.9
Df = 10
P < 0.0056

## Slide 6
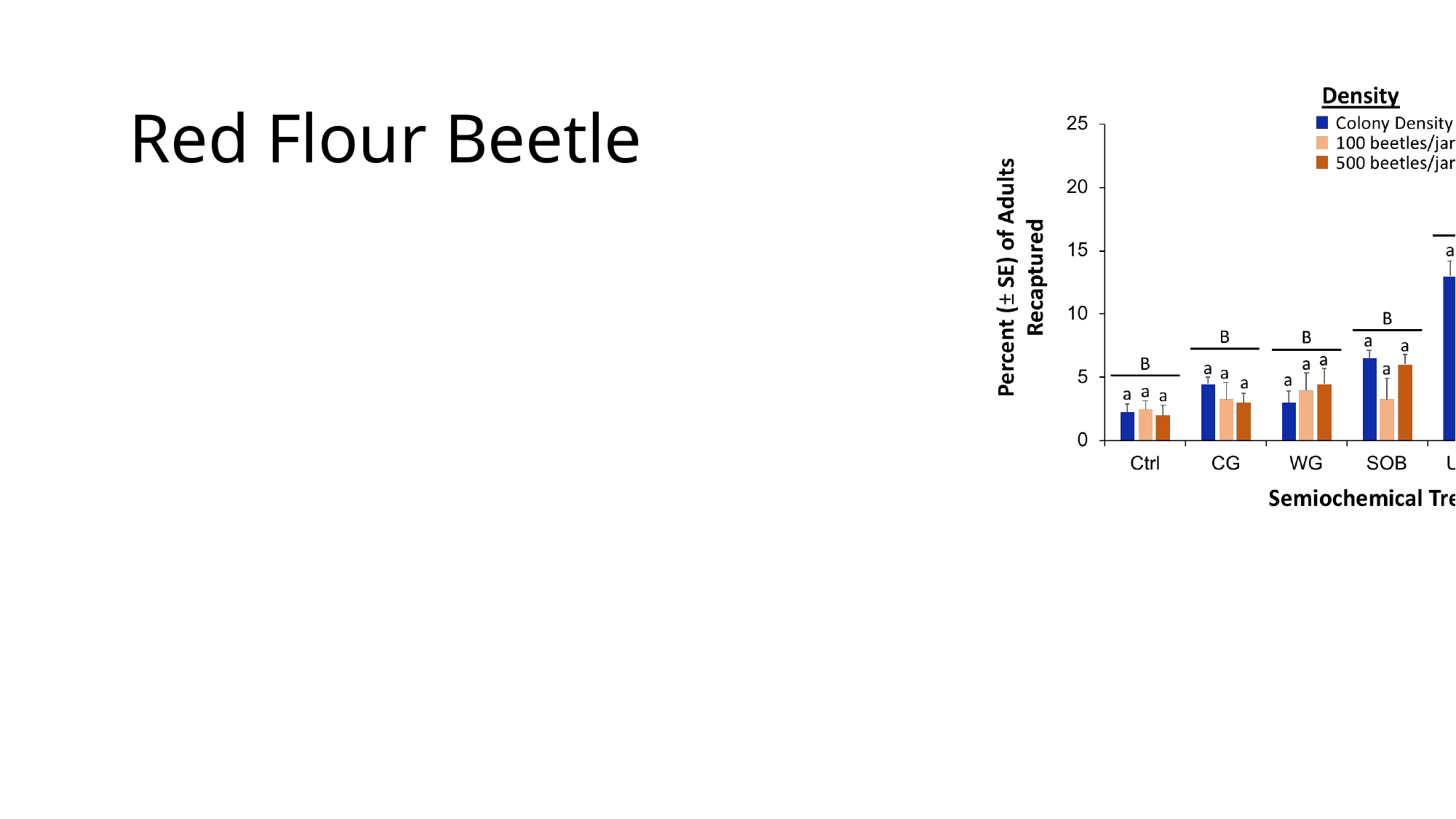

Red Flour Beetle

## Slide 7
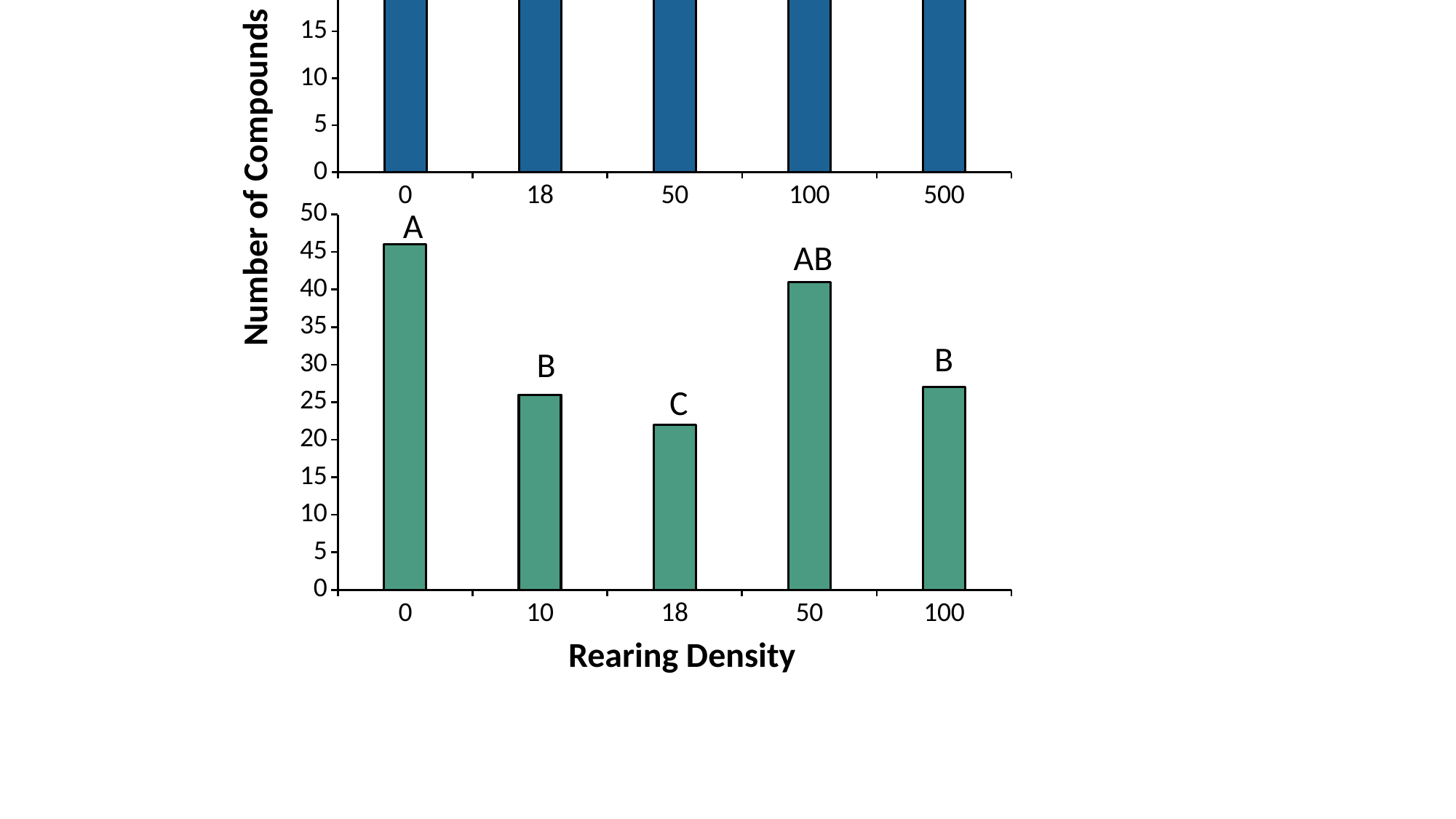

Species
T. castaneum
R. dominica
A
### Chart
| Category | |
|---|---|
| 0 | 36.0 |
| 18 | 28.0 |
| 50 | 29.0 |
| 100 | 38.0 |
| 500 | 32.0 |A
A
A
A
Number of Compounds
### Chart
| Category | |
|---|---|
| 0 | 46.0 |
| 10 | 26.0 |
| 18 | 22.0 |
| 50 | 41.0 |
| 100 | 27.0 |A
AB
B
B
C
Rearing Density

## Slide 8
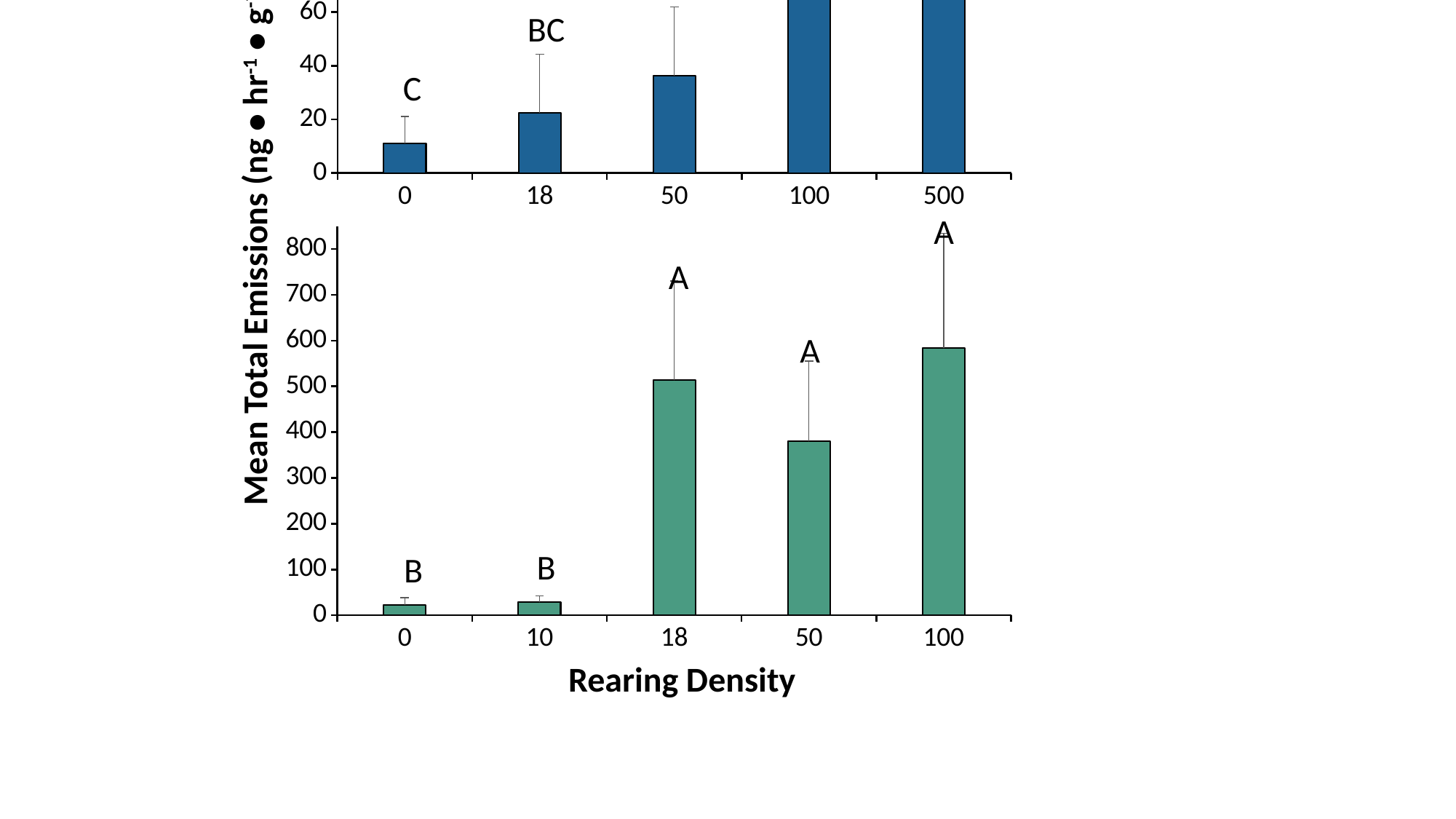

Species
T. castaneum
R. dominica
### Chart
| Category | |
|---|---|
| 0 | 11.145439234617022 |
| 18 | 22.43820604324162 |
| 50 | 36.180030192932506 |
| 100 | 78.34054203981246 |
| 500 | 118.18563 |A
AB
BC
BC
C
Mean Total Emissions (ng • hr-1 • g-1 wheat)
A
### Chart
| Category | |
|---|---|
| 0 | 22.60667550446681 |
| 10 | 28.13336415231559 |
| 18 | 513.7397745026224 |
| 50 | 380.3093055470367 |
| 100 | 584.5346023729609 |A
A
B
B
Rearing Density
